# Internet-based self-help therapy with FearFighter™ versus no intervention for anxiety disorders in adults: study protocol for a randomised controlled trial

**DOI:** 10.1186/s13063-016-1619-3

**Published:** 2016-10-28

**Authors:** Morten Fenger, Jane Lindschou, Christian Gluud, Per Winkel, Lise Jørgensen, Sten Kruse-Blinkenberg, Marianne Lau

**Affiliations:** 1 Stolpegaard Psychotherapy Centre, Mental Health Services, Capital Region of Denmark, Stolpegaardsvej 20, 2820 Gentofte, Denmark; 2 The Copenhagen Trial Unit, Centre for Clinical Intervention Research, Department 7812, Rigshospitalet, Copenhagen University Hospital, 2100 Copenhagen, Denmark; 3 Student Counselling Service, Dronningens Tværgade 30, 2, 1302 Copenhagen, Denmark

**Keywords:** Anxiety, Randomised feasibility trial, Cognitive behavioural therapy, Internet-based psychotherapy, FearFighter™

## Abstract

**Background:**

Internet-based self-help psychotherapy (IBT) could be an important alternative or supplement to ordinary face-to-face therapy. The findings of randomised controlled trials indicate that the effects of various IBT programmes for anxiety disorders seem better than no intervention and in some instances are equivalent to usual therapy. In Denmark, IBT is part of future treatment plans in mental health care services, but the verification level of the current clinical scientific knowledge is insufficient. The objective of this trial is feasibility assessment of benefits and harms of the Internet-based cognitive behavioural therapy (ICBT) programme FearFighter™ versus no intervention for anxiety disorders in adults.

**Methods and design:**

We will conduct an investigator-initiated, feasibility randomised controlled trial. Sixty-four participants are expected to be recruited via an advertisement posted on the homepage of the Student Counselling Service in Denmark. The inclusion criterion for participation in the trial will be the presence of anxiety disorder as assessed with the Mini International Neuropsychiatric Interview. The exclusion criteria will be suicidal risk, an ongoing episode of bipolar disorder or psychosis, concurrent psychological treatment for the anxiety disorder, considered unable to attend the intervention as planned (due to vacation, work/study placement, sickness, or similar occurrences), or lack of informed consent. The intervention group will be offered nine sessions with the ICBT programme FearFighter™ and a weekly telephone contact to support compliance. The control group will receive no intervention. We define the feasibility outcomes as follows: the fraction of randomised participants out of the eligible people (the lower 95 % confidence interval (CI) ≥ 50 %); and the fraction of compliant participants (those receiving at least six out of nine sessions) in the intervention group (the lower 95 % CI ≥ 60 %). The exploratory clinical outcomes are the number of participants no longer meeting the diagnostic criteria for an anxiety disorder at the end of the trial and level of distress (Beck Anxiety Inventory, Symptom Checklist-90-R, WHO Well-Being Index, Sheehan Disability Scale); the number of severe adverse events; and the occurrence of any psychological treatment outside the trial.

To prevent bias in design, and in the gathering and analysis of data throughout the trial, we will follow the SPIRIT 2013 statement which defines standard protocol items for clinical trials.

**Discussion:**

Based on our findings, we will discuss the feasibility of a future randomised controlled trial examining the benefits and harms of FearFighter™ versus no intervention for anxiety disorders in adults.

**Trial registration:**

ClinicalTrials.gov Identifier: NCT02499055, registered on 1 July 2015.

## Background

Anxiety disorders are characterised by excessive worries and fear of situations, objects, and living creatures. Anxiety disorders can be categorised into generalised anxiety disorder, social phobia, panic disorder, agoraphobia, specific phobias, post-traumatic stress disorder, and obsessive-compulsive disorder [1]. Reviews show that between 14 and 18 % of European citizens fulfil the diagnostic criteria for anxiety disorder according to the *Diagnostic and Statistical Manual of Mental Disorders* (DSM)-III, DSM-IV, or the *International Classification of Diseases, version 10* (ICD-10) [2, 3]. In Denmark, about 350,000 people may be suffering from an anxiety-related disorder [4]. Relatively few people are diagnosed and referred to treatment. In 2005, only 5976 people (under 2 % of the risk group population) were diagnosed with an anxiety disorder and received treatment in Denmark [4].

Cognitive behavioural therapy (CBT) appears to be efficacious in the treatment of anxiety [5] and is the recommended first-choice treatment both by the English National Institute of Health and Care Excellence (NICE) [6] and the Danish Health and Medicines Authority [7]. However, the availability of health care professionals is scarce and cannot cover the demand for traditional face-to-face psychotherapeutic treatment [4]. Therefore, ways for improving the availability of CBT are in demand. A way to save resources is to disseminate psychiatric treatment via different self-administered interventions such as Internet-based self-help psychotherapy programmes [8]. The latest meta-analysis assessing Internet-based self-help therapy (IBT) for anxiety disorders suggests that IBT has a moderate effect size (ES) of 0.79 standardised mean difference (SMD)^1^ on any self-rated symptom measure of anxiety compared with the waitlist control group [9]. IBT compared with face-to-face therapy suggests an ES of 0.05 SMD in favour of face-to-face therapy [9]. With the 2013 revision of the NICE guidelines for anxiety disorders, interventions with self-help resources in England are now recommended in line with face-to-face psychotherapy and pharmacological treatment using a stepped care model for anxiety [8, 10, 11]. In Denmark, IBT is sketched out as part of the future treatment in mental health care services [12, 13], but the current research results are still insufficient [14].

IBT for anxiety disorders is based on the same philosophy as face-to-face CBT and uses the same treatment principles. CBT is a highly structured and standardised treatment [15, 16]. Like CBT treatment manuals a standard Internet-based cognitive behavioral therapy (ICBT) programme consist of 6 to 12 sessions to be completed over 6 to 12 weeks. Each session in ICBT and in CBT contains the same four elements: psychoeducation, exercises and exposure to anxiety-provoking situations, homework (exercises/exposure), and measurement of symptoms. Psychoeducation is prioritised at the beginning of ICBT and CBT treatment programmes, while treatment exposure to anxiety-provoking situations is targeted towards the end.

FearFighter™ is an ICBT programme for the treatment of panic and phobia and it is used in the mental health services in England [8]. Two randomised trials from England have assessed FearFighter™ for patients with an anxiety disorder [17, 18]. Marks and colleagues compared FearFighter™ versus face-to-face behavioural therapy and versus computer-assisted relaxation. Intervention with FearFighter™ was superior to relaxation regarding self-rated anxiety symptoms, while FearFighter™ was equal to face-to-face behavioural therapy [17]. Schneider and colleagues tested FearFighter™ versus Internet-guided minimal CBT plus relaxation for patients with anxiety. No significant difference was found in terms of anxiety severity score between the two interventions at the end of treatment week 10. However, at week 14, significant differences were observed in symptom reduction in five out of ten outcomes favouring FearFighter™ [18].

In Denmark, one randomised trial with FearFighter™ was conducted and published as a nonpeer-reviewed report [19]. The trial compared FearFighter™ with a waitlist control group. The authors found no significant difference between the two groups on the Beck Anxiety Inventory (BAI). The authors reported that due to the chosen eligibility criteria, recruitment was poor and the dropout fraction was high: 50 % in the intervention group and 20 % in the waitlist group. Thus, the trial failed to produce meaningful results. The authors recommended that a new randomised controlled trial be conducted [19]. The objective of the present randomised controlled trial is to assess the feasibility and observe the efficacy of FearFighter™ versus no intervention in people with an anxiety disorder in Denmark.

## Methods/design

### Trial design

**Fig. 1:**
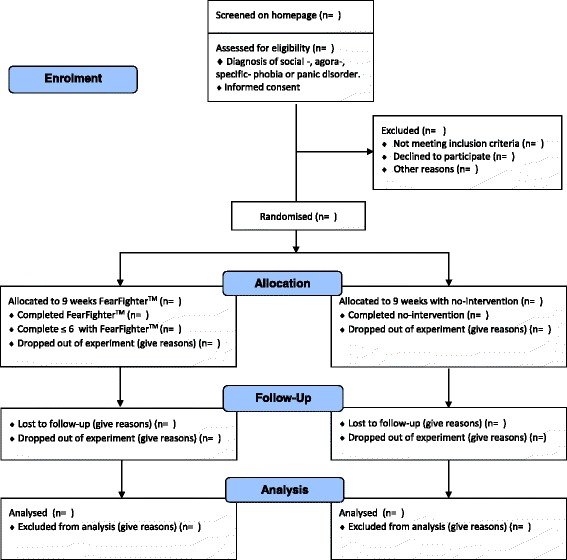
Consolidated Standards of Reporting Trials (CONSORT) trial flow chart for Internet-based self-help therapy with FearFighter™ versus no intervention for anxiety disorders in adults

The trial is an investigator-initiated, feasibility randomised controlled trial investigating ICBT with FearFighter™ compared with no intervention for adults with an anxiety disorder. We have followed the Standardised Protocol Interventions: Recommendations for Interventional Trials (SPIRIT) 2013 Statement which defines standard protocol items for clinical trials [20]. The Copenhagen Trial Unit (CTU) is responsible for the centralised randomisation. The randomisation is carried out according to a web-based computer-generated allocation sequence with varying block sizes kept unknown to the investigators. Once a participant is assessed as eligible for the trial the trial secretary will use the web-based randomisation system to allocate the participant to an intervention group. The allocation ratio is 1:1. Based on evidence of prognostic factors, the randomisation is stratified into: (1) social phobia and panic with agoraphobia, and (2) panic disorder without agoraphobia and specific phobia [21, 22]. Participants with panic disorder without agoraphobia and specific phobia have a distinctively higher remission rates than other types of anxiety [21, 22]. Assessment will be conducted prior to randomisation and start of intervention (week 0), at post-intervention (week 10), and at follow-up (week 37) (see the flow chart in Figure 1 in the Appendix). The trial has been approved by the Regional Ethical Committee for the Capital Region of Denmark (journal number: H-15005836), and by the Danish Data Protection Agency (journal number; RHP-2015-009 I-Suite 03652). The trial is registered at ClinicalTrials.gov at the United States National Institutes of Health (Identifier: NCT02499055, registered on 1 July 2015).

### Blinding

The trial participants, the support person for FearFighter™, and the administrators of FearFighter™ will be aware of the allocation. Baseline assessment will be conducted before participant randomisation. The assessment of symptoms and recovery at the final interview and at follow-up will be performed by blinded assessors with no knowledge of participant allocation.

Participants will be instructed to withhold information of their allocation group when assessed. Statistical analysis will be conducted blinded with the two intervention groups coded as, e.g. X and Y. Two abstracts with conclusions drawn will be prepared by the blinded investigators, one assuming that X is the experimental group and Y the control group, and one conclusion assuming the opposite [23, 24]. After this, the code will be broken. Analyses and conclusions regarding compliance cannot be blinded and will be kept separate.

### Study population

The study population will consist of adults with anxiety disorders with the following criteria for inclusion: age 18 years or older with panic disorder, specific phobia, agoraphobia, or social phobia according to the DSM-IV [25] as assessed with the Mini International Neuropsychiatric Interview (MINI) [26], and having given signed informed consent. Criteria for exclusion will be suicidal risk, an ongoing episode of bipolar disorder or psychosis, concurrent psychological treatment for the anxiety disorder, considered unable to attend the intervention as planned (due to vacation, work/study placement, sickness, or similar occurrences), or lack of informed consent.

### Sample size

The primary feasibility outcome is the number of randomised participants divided by the number of all eligible people for the trial. Eligible people are those who fulfil the inclusion criteria of our trial. If the number of randomised participants is 64 out of 100 eligible people then the proportion will be 64 % with 95 % confidence interval (CI) between 50 and 78 %. A proportion of 50 % or more randomised participants will be acceptable for a future trial. In contrast, if the likely sample proportion is less than 50 %, we will have difficulties with recruiting participants for a large trial as well as with generalisability of results.

### Recruitment and procedure

Participants are recruited via advertisements through the Danish Student Counselling Service and their website (www.srg.dk) with a link to our trial website. Our trial website contains information about the trial and a Contact Form for prospective trial participants. People visiting our trial website are asked to answer three screening questions from the Improving Access to Psychological Therapies (IAPT) Phobia Scale about how much they would avoid three different anxiety-provoking situations [27]. People who screen positive for a minimum of one of the three anxiety symptoms on the IAPT Phobia Scale are invited to sign up for an initial interview. A psychologist (the support person for FearFighter™) conducts the initial interview face-to-face (see Table 1 for an overview). Danish Regional Ethical Committee-approved written information about the trial is presented to interested people alongside with information about alternative places to receive advice and treatment for their anxiety. The interested people are advised not to participate in the trial if they need help urgently or if they consider the duration of the trial to be too long. The people are subsequently assessed with the use of the MINI and the Standardised Assessment of Personality Abbreviated Scale (SAPAS). The MINI is a diagnostic instrument for major axis I psychiatric disorders and is used to identify people with an anxiety disorder and the presence of comorbidity. As the MINI does not identify or screen for personality disorders, the SAPAS is used as a screening instrument [28]. The SAPAS will only be used for background information to indicate the possible presence of comorbid personality disorders in a person. People will be eligible as participants if they comply with the inclusion and exclusion criteria. If a person gives their written informed consent to become a participant in the trial they will be randomised. All people are informed that they may withdraw their consent at any time during the trial.

**Table 1:** Assessment tests and times of data collection in the FearFighter™ feasibility trial

Name of test	Screening/ website	Initial trial interview (week 0)	During trial	End-of-trial interview (week 10)	Follow-up (week 37)
Phobia Scale^a^	Xe		Xe		
Sociodemographic data		Xp			
Mini International Neuropsychiatric Interview		Xp		Xp	Xp
Standardised Assessment of Personality Abbreviated Scale		Xp			
Beck Anxiety Inventory		Xe		Xe	Xe
Symptom Checklist-90-R		Xe		Xe	Xe
WHO Well-Being Index		Xe		Xe	Xe
Sheehan Disability Scale		Xe		Xe	Xe
Adverse events			Xp	Xp	
Behaviour log			Xe		

*Xe* electronic collection of data, *Xp* paper collection of data

*WHO* World Health Organisation

^a^Improving Access to Psychological Therapies

### Data collection

Table 1 gives an overview of assessment tests and the time of data collection.

### Interventions

The participants in the experimental group receive Internet-based therapy using the programme FearFighter™ developed by Professor Isaac Marks from the Maudsley Psychiatric Hospital in England [29]. FearFighter™ is a commercial online programme for the treatment of panic and phobia [30]. A Danish version of the programme was developed in 2013 [19]. The FearFighter™ programme is based on principles derived from CBT. The programme aims to teach people how to tackle negative thoughts and to challenge avoidance behaviours due to anxiety disorders. Participants receive an email with a link and an activation code for FearFighter™ as well as an empty ring binder to organise printed documents and session summaries from FearFighter™.

FearFighter™ consists of nine sessions. In each session a video-filmed therapist gives psychoeducation and explains the rationale for the training, followed by a number of exercises and tasks for homework. A whole week is assigned to do the homework. Homework is done between each session, and it is estimated to take 1 to 3 h, depending on the invested effort by the individual. Each on-line session excluding the homework takes about 30 to 40 min. The content of the nine sessions is described below.

#### Session 1

This consists of psychoeducation about CBT for anxiety, and the programme. The homework is to find a person who can encourage the patient to complete the sessions in the programme.

#### Session 2

This consists of psychoeducation about anxiety symptoms and safety behaviour. The homework is to register episodes of anxiety in an electronic diary built within the programme.

#### Session 3

This consists of psychoeducation about panic disorder and interoceptive exposure. Demonstration of three interoceptive exercises: flash cards, progressive relaxation, and applied tension. The homework is to do one or more of the exercises on a daily basis.

#### Session 4

This consists of psychoeducation about automatic negative thoughts. The homework is to challenge the negative thoughts with rational questions and to write answers and thoughts in a schema.

#### Session 5

This consists of psychoeducation about core beliefs about oneself. The homework is to challenge the negative core beliefs with rational questions and to try to replace them with positive core beliefs.

#### Session 6

This consists of psychoeducation about exposure and the hierarchy model of anxiety-provoking situations. The homework is to make a hierarchy of anxiety-provoking situations including goal setting.

#### Session 7

This consists of psychoeducation about how to prepare for exposure by pictures, sounds, and video. The homework is to prepare the exposure plan.

#### Session 8

This consists of support for the patient in the exposure exercises. The exposure exercises have to be repeated until the patient fulfils their exposure plan and goal setting.

#### Session 9

This session involves summing-up and counselling on how to master setbacks and prevent relapse and suggests continuing exposure exercises if necessary.

Between the sessions the programme will ask the person to indicate how bothered they are by their symptoms. The programme uses five brief questionnaires from IAPT about: (1) depression, (2) generalised anxiety, (3) phobia, (4) work and social adjustment, and (5) self-harm. The system gives feedback to the participant, showing a graph on treatment progress. In all, the FearFighter™ programme recommends that participants spend 5 to 8 h with the FearFighter™ programme and spend 32 to 35 h practising the assignments and exposure exercises.

### Support person and support manual

Each trial participant is contacted over the telephone by a support person once a week during the 9 weeks of intervention. Each telephone contact should take about 10 min and a maximum of 1.5 h for the whole intervention period. The purpose of the contact is to secure and assess compliance and also to assist the participant’s adherence to the programme. The support person follows a support manual for the amount, type, and content of the support given. The support person must not engage in a psychotherapeutic dialogue with the participants. The support person who performs all the initial interviews and baseline assessments will also support the 32 participants in the experimental group. The reason for this is to optimise the alliance between the participants and the support person.

The manual for the support person prepared by the company behind the Danish version of FearFighter™ was tested by us, the trial investigators, and found to be too unspecific and vague for having standardised support in our trial. Therefore, we prepared our own manual with specific instructions for the support at each session in FearFighter™. None of the instructions in our manual are in conflict with, or violate the general instructions in, the support manual by the Danish company. Both the original support manual and our manual are written in Danish and can be obtained upon request.

### Assessors

The protocol coauthor LJ (MSc in psychology), is trained by a senior psychiatrist in conducting the MINI. LJ trains and instructs the post-treatment and follow-up assessors (graduate students in psychology) in the use of the MINI in order to secure reliability in the assessments. Although preferable, no interrater reliability measurement will be performed.

## Outcomes

### Feasibility outcomes

The primary feasibility outcome is the proportion of randomised participants out of all eligible people. Eligible participants are those who fulfil our inclusion criteria. The secondary feasibility outcome is compliance, defined as the number of participants completing at least six of the nine planned FearFighter™ sessions in the experimental group. FearFighter™ will automatically register time for login and save the exercises that have been completed. The support person sees a tracking log on the administration webpage for FearFighter™ for each participant and the compliance will be assessed during the weekly telephone calls with the experimental participants and summed-up at the end of the trial interview.

Participants who complete six or more sessions in FearFighter™ but who fail to show up for post-treatment assessment are still regarded as completers in the feasibility outcome. If the participants complete less than six sessions they will be regarded as noncompleters (dropouts).

### Exploratory clinical outcomes

The primary exploratory clinical outcome is the proportion of participants who no longer fulfil the diagnostic criteria for an anxiety disorder at the end of the intervention as assessed with the MINI [26]. Other exploratory clinical outcomes are severity of psychiatric symptoms, disability, and well-being measured using the following described participant-reported instruments: the Beck Anxiety Inventory (BAI) is a 21-item general questionnaire for anxiety symptoms during the last 14 days rated on a 4-point Likert scale from 0 to 3. The maximum score is 63. A score of 26 or greater indicates severe anxiety [31]. The Symptom Checklist-90 revised (SCL-90-R) is a 90-item multidimensional questionnaire measuring psychopathology and psychological distress during the last 7 days on nine primary symptom dimensions and three global dimensions. Each item is rated on a 5-point Likert scale from 0 to 4. The score on each dimension is the mean of the included items [32]. The Global Severity Index (GSI), interpersonal sensitivity dimension (IS), anxiety dimension (ANX), and phobic anxiety dimension (PHOB) are used in this trial. The SCL-90-R is a valid and reliable outcome, used often in psychotherapy research, and Danish norms are available [33]. The Sheehan Disability Scale (SDS) is a 3-item questionnaire for occupational function, social function, and family function rated on an 11-point Likert scale. Specific scoring for the three functions is from 0 to 10 and a global score for the general function is from 0 to 30. A score of 5 or greater on the specific scales indicates dysfunction [34]. The WHO Well-Being Index is a 5-item questionnaire for the assessment of health-related quality of life for the last 14 days on a 6-point Likert scale from 0 to 5. Scores are summated, with raw scores ranging from 0 to 25 [35].

Serious adverse events and other untoward events that require hospitalisation, are life-threatening, or result in death are collected following the SPIRIT recommendations [20]. All adverse and untoward events will be categorised and summated. Finally, the proportion in the groups of experimental and control participants who are compliant with the randomised intervention, defined as the lack of any psychological treatment during the 9-week intervention period, will be registered. At follow-up, it will be registered if participants have been engaged in psychological treatment since the end of the trial, although no restrictions will be imposed on the participants during the follow-up period.

All scores will be compared between the two groups at post-treatment (week 10) and at follow-up (week 37). Serious adverse events and other untoward events will be gathered during the intervention for the intervention group and at post-treatment for both the control and the intervention groups.

### Statistical analyses

The primary feasibility outcome is defined as the number of participants randomised out of the people considered eligible for being randomised in the trial. Based on observations regarding pretreatment attrition in CBT and IBT for anxiety disorders studies [9, 36–38], we will consider the feasibility outcome satisfactory if it is over 50 %. Our hypothesis is that we are sampling from a population of eligible people of whom at least half will be randomised. If we let *N* be the observed number of eligible people needed to randomise a sample of 64 participants, then in a random sample of size *n* sampled from a population sized *N*, *n/N* follows a binomial distribution with a lower 95 % CI of over 50 % [39, 40]. The sample size estimated from the primary feasibility outcome implicitly defines the sample sizes to be used in the assessment of the exploratory clinical outcomes (Table 1).

The secondary feasibility outcome for the participants in the experimental group is defined as the number of participants completing at least six out of nine FearFighter™ sessions. This feasibility outcome is considered satisfactory when the lower 95 % CI is over 60 % based on observations for completer fractions in IBT for anxiety disorders [9, 41]. In a random sample of size *n* of completers sampled from a population sized *N* of participants in the experimental group, *n*/*N* follows a binomial distribution with a lower 95 % CI over 60 %.

The exploratory clinical outcomes will be analysed using the general linear model (GLM) logistic regression, or the proportional odds model for ordinal outcomes as appropriate, adjusting for the stratification variables and baseline scores of the outcomes according to Table 2. We will calculate two-sided tests and use the resulting *p* values as a data-reducing device because test results with *p* values < 0.05 will be used to select hypothesis-generating outcomes.

**Table 2:** Feasibility outcomes and exploratory clinical outcomes in the Fearfighter™ feasibility trial

	Rating scales	Type of outcome	Test
Feasibility outcome			
Number of randomised participants/eligible persons	Not relevant	Proportion	Calculate the probability that 64/*N* can have been obtained in a random sample of *N* participants obtained from a population of persons where half the eligible persons can be randomised. (*N* is the observed number of eligible persons necessary to examine to randomise 64 participants)
Number of participants complying at least 6/9 FearFighter™ sessions/ randomised participants	Not relevant	Proportion	Test analogous to the above feasibility outcome with the difference that now we want to secure that the sample is not from a population being less than 60 % compliant
Exploratory outcome			
Number of participants who no longer fulfil the diagnostic criteria for an anxiety disorder	MINI	Proportion	Fisher’s exact test and logistic regression adjusted by the protocol-specified stratification variable (the result of the latter test is the primary of the 2 tests)
Severity of anxiety	BAI	Continuous	The general linear univariate model (GLM) taking stratification variables and baseline BAI into account
Psychological distress and psychopathology	SCL-90-R	Continuous	GLM taking stratification variables and baseline SCL-90-R into account
Positive well-being	WHO-5	Ordinal	The proportional odds model for ordinal response taking stratification variables and baseline WHO-5 into account (if conditions are not fulfilled, then nonparametric Mann-Whitney)
Functional impairment	SDS	Continuous	GLM taking stratification variables and baseline SDS into account.
Number of randomised receiving psychological treatment during the 9 weeks	Interview	Proportion	Fisher’s exact test and logistic regression adjusted by the protocol-specified stratification variable (the result of the latter test is the primary of the 2 tests).
Number of participants with one or more adverse event	Interview	Proportion	Fisher’s exact test and logistic regression adjusted by the protocol-specified stratification variable (the result of the latter test is the primary of the 2 tests)

*BAI* Beck Anxiety Inventory, *MINI* Mini International Neuropsychiatric Interview*, SCL-90-R* Symptom Checklist-90 revised, *SDS* Sheehan Disability Scale, *WHO* World Health Organisation

We have added one additional exploratory outcome, namely the proportion of participants in the experimental and control groups who are compliant with the randomised intervention, defined as the absence of any psychological treatment during the 9-week intervention period. This outcome, as well as the proportion of participants experiencing an adverse event, will be analysed as the other exploratory outcomes with Fisher’s exact test and logistic regression, adjusted by the protocol-specified stratification variable (the result of the latter test is the primary of the two tests). Adverse events will also be reported according to type, severity, and probable relation to the interventions for the two intervention groups.

## Discussion

The aim of our trial is to investigate the feasibility and observe the efficacy of the ICBT programme FearFighter™ for people with anxiety disorders in Denmark. We expect to widen the scope of this relatively new area of research on Internet-based therapy as the conducted Danish trial on FearFighter™ demonstration project failed to recruit the intended number of participants and suffered from a participant dropout rate of 50 % in the intervention group [19].

Our trial is being conducted in cooperation with the CTU, a centre for clinical intervention research. The design of this trial is in accordance with the guidelines provided by the SPIRIT Statement [20] and the standard operating procedures, developed and maintained by the CTU in order to secure the validity and reliability of the trial results [42]. We have tried our utmost to reduce risks of bias [23, 24, 43–47] by employing centralised randomisation, blinding of outcome assessors, blinding during data management, blinded statistical analyses, and blinded drawing of conclusions to fulfil national and international standards of Good Clinical Practice [48].

Our criteria for inclusion and exclusion are few because we believe that the results of our trial will have a potentially wide generalisability. It should be also noted that the participants will be recruited among visitors to the Student Counselling Service’s homepage. Therefore, we may expect that the participants, students from Danish universities suffering from an anxiety disorder, to be rather homogenous concerning duration and severity of symptoms, age, cultural and economic background, and a low prevalence of comorbidity, which otherwise may limit the generalisability of the results to more clinically heterogeneous samples.

We will consider our feasibility trial sufficiently successful if more than 50 % of the eligible participants are randomised and if more than 60 % of the 32 randomised participants complete FearFighter™. A satisfactory feasibility outcome value will warrant a future large-scale trial to investigate further the observed efficacy and the cost-benefit of FearFighter™.

### Trial status

This trial will begin recruiting participants in August 2015. The last participant of the 64 participants will be included in May 2016. We estimate to have collected post-treatment data by summer 2016 and follow-up data by winter 2016.

## Abbreviations

ANX: Anxiety dimension
BAI: Beck Anxiety Inventory
CBT: Cognitive behavioural therapy
CI: Confidence interval
DSM-IV: *Diagnostic and Statistical Manual of Mental Disorders – 4th edition*
ES: Effect size
GSI: Global Severity Index
IAPT: Improving Access to Psychological Therapies
IBT: Internet-based self-help therapy
ICBT: Internet-based cognitive behavioural therapy
IS: Interpersonal sensitivity dimension
M: Mean
MINI: Mini International Neuropsychiatric Interview
*N*: Number in population
*n*: Number in sample
NICE: National Institute of Health and Care Excellence
PHOB: phobic anxiety dimension
RCT: Randomised controlled trial
SAPAS: Standardised Assessment of Personality Abbreviated Scale
SCL-90-R: Symptom Checklist-90 revised
SD: Standard deviation
SDS: Sheehan Disability Scale
SMD: Standardised mean difference
SPIRIT: Standard Protocol Items: Recommendations for Interventional Trials
WHO: World Health Organisation

## Footnotes

1 An index of effect equivalent to Cohen’s *d*.
